# Estrogen, Epigenetics, and Cardiometabolic Health: Mechanisms and Therapeutic Strategies in Postmenopausal Women

**DOI:** 10.3390/cells15060529

**Published:** 2026-03-16

**Authors:** Ailene Edwards, Pranjal Singh, Vyan Shah, Vivek Chander, Sumita Mishra

**Affiliations:** 1 Center for Exercise Medicine Research, Fralin Biomedical Research Institute, Virginia Tech Carilion, Roanoke, VA 24016, USA; 2 Department of Surgery, Virginia Tech Carilion School of Medicine, Roanoke, VA 24016, USA; 3 Department of Human Nutrition, Foods, and Exercise, College of Agriculture and Life Sciences, Virginia Tech, Blacksburg, VA 24061, USA; 4 Center for Vascular and Heart Research, Fralin Biomedical Research Institute, Virginia Tech Carilion, Roanoke, VA 24016, USA

**Keywords:** estrogen signaling, epigenetic regulation, cardiometabolic health, postmenopausal women, hormone replacement therapy

## Abstract

The loss of estrogen following menopause is associated with a marked increase in cardiometabolic risk, accompanied by adverse changes in lipid metabolism, insulin sensitivity, vascular function, and systemic inflammatory tone. Emerging evidence suggests that estrogen signaling interacts with chromatin regulatory mechanisms, including DNA methylation, histone modifications, and chromatin remodeling, across multiple metabolic tissues. In this review, we examine current evidence linking estrogen receptor signaling to epigenetic modulation in cardiovascular, hepatic, adipose, vascular, and immune systems. We propose that epigenetic remodeling represents a plausible and testable mechanistic framework connecting estrogen depletion to cardiometabolic disease progression, while acknowledging that much of the mechanistic evidence derives from preclinical and in vitro systems and that direct longitudinal validation in human cardiovascular tissues remains limited. We further explore how this framework may contribute to understanding the “estrogen paradox” and the heterogeneous outcomes of hormone replacement therapy (HRT), particularly within the context of the timing hypothesis. Finally, we evaluate pharmacologic and lifestyle interventions, including structured exercise, dietary modulation, and cardiometabolic therapeutics, through the lens of potential epigenetic influence. Clarifying tissue-specific and immune-integrated chromatin responses to estrogen loss will be essential for advancing precision strategies aimed at improving cardiometabolic health in postmenopausal women.

## 1. Introduction

Aging in women is characterized by a series of programmed hormonal changes that critically impact systemic metabolic and cardiovascular health. The transition from reproductive years to the final postmenopausal state is a gradual, multi-year continuum rather than an abrupt event. The Stages of Reproductive Aging Workshops (STRAW) framework provides the definitive clinical and endocrinological staging system for this transition, delineating three primary phases: the reproductive phase, the menopausal transition, and postmenopause [1,2] (Table 1). A progressive decline in ovarian follicles leads to reduced Inhibin B and anti-Müllerian hormone (AMH) levels, resulting in elevated follicle-stimulating hormone (FSH) and increasingly erratic estrogen secretion [3,4,5,6,7]. This hormonal instability ultimately culminates in the chronically low, stable estrogen state that defines postmenopause, driving widespread metabolic adaptations and increasing disease susceptibility [2,3,4,5]. The loss of estrogen in postmenopausal women is associated with increased risks of cardiovascular disease (CVD), metabolic syndrome, and mental health disorders [2,3,4,5,6,7]. The withdrawal of estrogen’s protective regulatory effects promotes a systemic shift toward a pro-inflammatory, oxidative, and insulin-resistant state. This review examines the molecular mechanisms underpinning this transition, positing that epigenetic reprogramming represents a plausible and testable hypothesis linking estrogen loss to the onset of cardiometabolic pathology. This hypothesis is supported by convergent preclinical evidence but requires direct validation in human cardiovascular tissues. It is also important to recognize that cardiometabolic risk after menopause is not solely attributable to chronological aging, as women with premature ovarian insufficiency demonstrate substantially elevated cardiovascular risk despite younger vascular age, supporting a direct protective role of estrogen in cardiovascular tissues.

## 2. The Estrogen Paradox

The “estrogen paradox” refers to the conflicting evidence surrounding estrogen’s role in cardiovascular protection and the contradictory outcomes observed in clinical studies. On one hand, extensive epidemiological and experimental evidence supports a “protective hypothesis.” Estrogen is widely recognized for its vasoprotective, anti-inflammatory, and metabolic-regulating properties, contributing to favorable lipid profiles, improved insulin sensitivity, and reduced vascular inflammation [8]. Clinical data show that, compared with age-matched premenopausal women, early onset of menopause is associated with a two- to six-fold-higher incidence of cardiovascular disease, whereas each additional year that menopause is delayed reduces this risk by approximately 3% [9]. On the other hand, large-scale randomized clinical trials evaluating hormone replacement therapy (HRT) for cardiovascular prevention—notably the Women’s Health Initiative (WHI) and the Heart and Estrogen/progestin Replacement Study (HERS)—failed to confirm the anticipated cardioprotective effects and in some cases reported neutral or even adverse cardiovascular outcomes [10]. This paradox underscores the complexity of estrogen signaling and its diverse effects on cardiovascular physiology. Current evidence suggests that the estrogen paradox is most parsimoniously explained by progressive vascular aging and plaque burden at the time of therapy initiation, changes in estrogen receptor subtype expression across tissues, route-dependent hepatic first-pass effects on coagulation pathways, and thrombotic risk modulation. In addition, emerging preclinical and associative evidence suggests that epigenetic remodeling, particularly time-dependent changes in estrogen receptor promoter methylation and chromatin accessibility, may contribute a complementary mechanistic layer to the timing hypothesis. However, this epigenetic contribution remains hypothesis-level and requires longitudinal human chromatin profiling data for confirmation.

### Estrogen Signaling Pathways: Impact on Cardiometabolic Health

**Figure 1:**
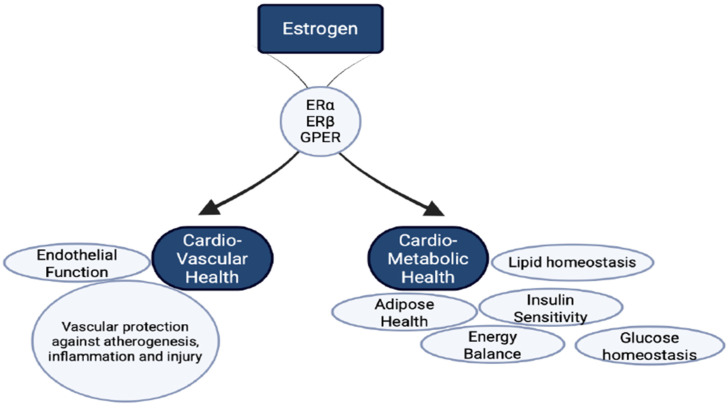
Estrogen receptor-mediated cardioprotective outcomes: This schematic diagram provides a conceptual overview of major physiological domains influenced by estrogen signaling through estrogen receptor alpha (ERα), estrogen receptor beta (ERβ), and the G-protein-coupled estrogen receptor (GPER/GPR30). Estrogen receptor signaling has been associated in observational and experimental studies with improved cardiovascular health by promoting endothelial function and providing vascular protection against inflammation, atherogenesis, and tissue injury. In parallel, estrogen signaling contributes to cardiometabolic regulation, influencing adipose tissue biology, lipid handling, insulin sensitivity, glucose homeostasis, and overall energy balance. Together, these pathways illustrate the broad protective roles of estrogen in maintaining vascular integrity and metabolic stability.

Estrogen signaling plays a central role in regulating lipid metabolism, fat distribution, and insulin sensitivity (Figure 1). With advancing age, the decline in estrogen disrupts these pathways and contributes to increased cardiometabolic risk. Estrone (E1), estradiol (E2), and estriol (E3) are the three forms of estrogen, with E2 being the most potent and predominant during reproductive years [11,12]. Although the ovaries are the primary source of estrogen, several peripheral tissues—including adipose tissue, the brain, and the cardiovascular system—also produce estrogen locally.

**Figure 2:**
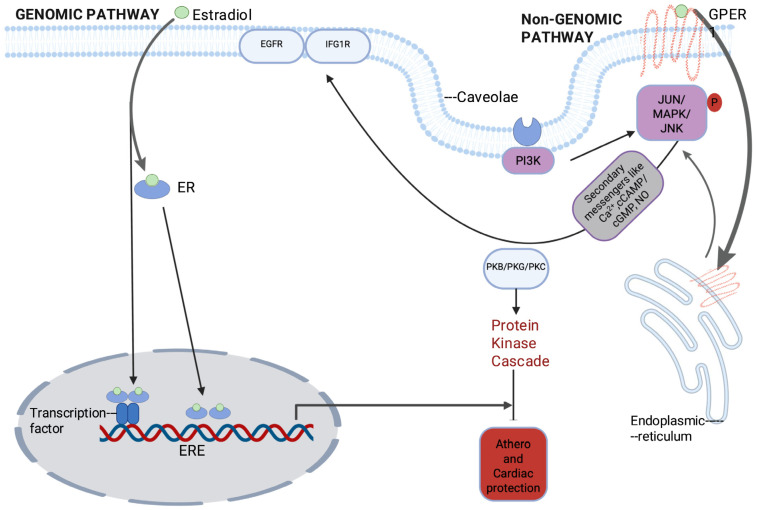
Estrogen receptor signaling pathways: Estrogen receptor (ER) signaling occurs through genomic and non-genomic mechanisms. In the genomic (classical) pathway, intracellular ERs are activated by ligands, of which 17β-estradiol (E2) is the most potent natural ligand. Estradiol binding induces ER dimerization and nuclear translocation; activated ER dimers bind directly to estrogen-response elements (EREs) or interact with other DNA-bound transcription factors to regulate target gene transcription. In non-genomic signaling, a membrane-associated pool of ERs, often localized within caveolae, rapidly initiates cytosolic signaling cascades that modulate protein phosphorylation and second-messenger systems. These pathways can transactivate receptor tyrosine kinases, including the epidermal growth factor receptor (EGFR) and insulin-like growth factor 1 receptor (IGF1R), leading to activation of phosphoinositide 3-kinase (PI3K) and downstream kinases such as protein kinase B (PKB/Akt), protein kinase G (PKG), and protein kinase C (PKC), thereby contributing to cardiovascular and metabolic regulatory effects. Estrogenic signaling also occurs via the G protein-coupled estrogen receptor (GPER1, also known as GPR30), located at the plasma membrane and intracellular membranes (e.g., endoplasmic reticulum). Estradiol binding to GPER1 activates PI3K and mitogen-activated protein kinases (MAPKs) signaling including c-Jun N-terminal kinases (JNKs) along with related downstream signaling intermediates.

Estrogen acts through three estrogen receptors (ERs): ERα, ERβ, and the G-protein-coupled estrogen receptor (GPER). These receptors mediate signaling via two major mechanism (Figure 2): Genomic (Classical) Signaling: ERα and ERβ function as ligand-activated nuclear receptors. Upon estrogen binding, they dimerize, translocate to the nucleus and regulate transcription by binding to estrogen response elements (EREs) [11,12]. ERs can also influence transcription indirectly by interacting with other transcription factors via protein–protein interactions [13]. Non-Genomic (Rapid) Signaling: A subset of ERs localize to the plasma membrane, where they rapidly activate intracellular signaling pathways such as ERK1/2 and PI3K [12,13]. Additionally, GPER mediates rapid cyclic-AMP (cAMP) production and Ca2+ mobilization, contributing to metabolic and vascular regulation [14].

## 3. Local Estrogen Biosynthesis and Cardiac Function

Local Estrogen Biosynthesis in the heart is primarily mediated by cytochrome P450 aromatase (CYP19A1), which converts androstenedione and testosterone into estrogens [15]. Expression of aromatase within cardiac tissue supports in situ estrogen production and suggests a direct influence on cardiomyocyte metabolism and energy homeostasis [16,17]. Genetic polymorphisms in CYP19A1 have been associated with CVD risk, implicating endogenous estrogen production in cardiometabolic regulation [18]. After menopause declining systemic estrogen levels are associated with increased obesity and higher incidence of type 2 diabetes mellitus (T2DM) [19,20]. At the transcriptional level, ERα has been shown, primarily in animal models and cardiomyocyte cell systems, to engage in crosstalk with peroxisome proliferator-activated receptor-α (PPARα), modulating pathways involved in cardiac hypertrophy and heart failure [21]. Estrogen-related receptor-α (ERRα) also governs cardiac energy metabolism through its interaction with peroxisome proliferator-activated receptor-γ coactivator 1α (PGC-1α) [22], raising the possibility that E2/ERα signaling can potentiate ERRα-driven metabolic programs [23]. The role of ERβ in metabolic control remains less clear, with conflicting reports regarding its effects on adiposity and lipid metabolism [24,25]. Nonetheless, ERβ has been proposed to modulate mitochondrial complex IV activity in the heart, helping preserve mitochondrial integrity and contributing to cardioprotection in the setting of trauma or stress [26].

## 4. Lipid Metabolism

Postmenopausal estrogen decline profoundly disrupts lipid homeostasis. Because estradiol (E2) synthesis utilizes low-density lipoprotein (LDL) cholesterol as substrate, reductions in E2 are associated with decreased LDL utilization [27]. ERα directly regulates hepatic genes involved in lipid metabolism, and impairment of ERα signaling promotes triglyceride accumulation within hepatocytes [28,29]. Estrogen also influences cholesterol handling through proprotein convertase subtilisin/kexin type 9 (PCSK9), which targets LDL receptor degradation. Estradiol suppresses PCSK9 expression and activity, thereby preserving LDL receptor function and lowering circulating LDL levels [30]. In the heart, GPER contributes to fatty acid metabolism by upregulating GCN5L1, a regulator of medium-chain acyl-CoA dehydrogenase activity that supports efficient mitochondrial β-oxidation [31]. The postmenopausal period further impairs liver metabolic function, with mitochondrial dysfunction, cellular senescence, and fibrosis collectively exacerbating lipid dysregulation. Transcriptomic analyses of aged livers reveal elevated reactive oxygen species (ROS), reduced ATP synthesis, and decreased NAD^+^ availability [32]. Consistent with this, PCSK9 expression also correlates with aging-related apoptotic and TNF-driven inflammatory pathways, and PCSK9 inhibition mitigates inflammatory and fibrotic remodelling in the liver while reducing oxidative stress and cardiac dysfunction [33]. Estrogen signaling through PPARα regulates cardiac free fatty acid oxidation. Ovariectomized diabetic mouse models—which mimic postmenopausal estrogen deficiency, exhibit marked lipid accumulation in cardiomyocytes, a phenotype ameliorated by GPER agonists that restore PPARα activity [34]. Additionally, postmenopausal aging is associated with increased PPARγ acetylation, which shifts its activity toward a more pro-atherogenic, pro-inflammatory lipid profile. In contrast, deacetylated PPARγ promotes improved lipid handling, lowers LDL levels, and exerts anti-inflammatory effects [35].

## 5. Fat Distribution

Aging leads to increased visceral adipose tissue (VAT), particularly after postmenopause, even when total fat mass is unchanged [7,36]. Estrogen’s role in fat distribution remains incompletely defined, in part because of methodological challenges in assessing ER activity in adipocytes. ERα appears to play a dominant role in regulating adiposity, as ERα knockout models develop marked metabolic dysfunction, whereas ERβ knockout mice do not show the same degree of disturbances [37]. Mechanistically, E2/ERα signaling induces sirtuin 1 (SIRT1) expression, which suppresses both adipogenesis and autophagy via the mTOR-ULK1 and STAT3-p55 pathways [38].

Chemokine receptors may also regulate adipocyte differentiation. Expression of the chemokine receptors CXCR2 and CXCR4 in adipocytes influences adipogenesis, with CXCR4 signaling downregulating ERα in proliferating cells [39,40]. Beyond chemokine signaling, proteomic analyses identify leptin and fatty acid-binding protein 4 (FABP4) as biomarkers correlating with body mass index (BMI) and waist-to-height ratio, whereas caspase-8 and cathepsin L1 are more specifically linked to visceral fat accumulation [41]. Senescent adipose cells secrete pro-inflammatory cytokines that promote visceral fat expansion and metabolic dysfunction [42,43]. Natriuretic peptides (NPs), such as atrial natriuretic peptide (ANP) and brain natriuretic peptide (BNP), further regulate fat metabolism and distribution. Estrogen modulates NP signaling, and its decline after menopause is associated with reduced NP levels, promoting visceral fat accumulation and impairing lipid mobilization [44]. NP signaling enhances lipid oxidation and promotes the browning of white adipose tissue, conferring cardiometabolic protection that diminishes with aging [45].

These hormonal changes also underlie the distinct fat-distribution patterns between men and women. Men predominantly store fat in the abdominal region (android or apple-shaped), with greater visceral fat accumulation that increases with age and declining testosterone. In contrast, premenopausal women store fat in the hips and thighs (gynoid or pear-shaped) because estrogen protects against visceral fat accumulation. With the menopausal transition, declining estrogen shifts fat deposition toward the abdominal region, increasing visceral fat and the risk of metabolic syndrome and cardiovascular disease [46].

## 6. Insulin Resistance

Aging and obesity drive insulin resistance, primarily through visceral adiposity [7,47]. Cellular senescence in adipose tissue exacerbates this process, with elevated senescence markers observed in T2DM states [48]. Compared with pre-menopausal females, postmenopausal females exhibit higher rates of insulin resistance, reflected by elevated fasting glycemic indices such as glucose, HbA1c, and GlycA [49]. Estrogen signaling via GPER supports pancreatic islet survival and glucose homeostasis. GPER knockout mice exhibit glucose intolerance and hyperglycemia due to impaired insulin release, whereas GPER agonists restore glucose balance and suppress inflammation in ovariectomized mice [50,51,52]. ERα also modulates insulin sensitivity by regulating glucose transporter type 4 (GLUT4) in adipose and skeletal muscle, both essential for glucose uptake. Loss of ERα diminishes GLUT4 expression, thereby exacerbating insulin resistance [29]. Estrogen further suppresses hepatic gluconeogenesis by inhibiting *Foxo1* transcription through PI3K-Akt signaling [53]. The NLRP3 inflammasome, linked to aging and cardiovascular disease, also contributes to insulin resistance through immune–epigenetic crosstalk; NLRP3 acetylation in macrophages and adipose tissue immune cells activates inflammatory signaling and impairs glucose metabolism, whereas deacetylation improves glucose homeostasis in aging models [54]. Mitochondrial dysfunction and ROS generation further aggravate insulin resistance, particularly in skeletal muscle, underscoring the need for additional in vivo studies [55].

### Estrogen’s Epigenetic Footprint on Cardiovascular Health

Evidence linking estrogen signaling to epigenetic regulation spans multiple tissues and experimental systems. Much of the mechanistic detail regarding AKT–DNMT–EZH2–KDM signaling derives from cancer biology, vascular smooth muscle cell studies, endothelial models, adipocyte progenitors, and murine systems. Direct in vivo human evidence demonstrating estrogen-dependent chromatin remodeling within cardiomyocytes remains comparatively sparse. Accordingly, the pathways described below are presented with distinction between validated findings and proposed mechanistic extensions. The key tissue-specific mechanisms, receptor subtypes, and evidence levels are summarized in (Table 2). The cumulative body of evidence underscores the pivotal role of ERs in cardiovascular health, primarily by inhibiting apoptosis [56], repressing hypertrophy [57], preventing atherosclerosis [58], reducing oxidative stress, and promoting angiogenesis [58]. These protective effects are intricately regulated by co-regulators, that function as either coactivators or corepressors. For example, Bcl3, identified as a coactivator of both ERRα and PPARα, facilitates transcriptional regulation by recruiting class I coactivators such as Steroid Receptor Coactivator-1 (SRC-1), which possesses histone acetyltransferase activity [59].

Beyond classical nuclear receptor activity, ERs also engage in non-genomic signaling. Palmitoylated ERα and GPER1, localized to the plasma membrane, where they interact with the p85 regulatory subunit of phosphatidylinositol 3-kinase (PI3K) within caveolae, leading to AKT activation [60,61,62] (Figure 2). AKT signaling has been reported in several cellular systems to influence chromatin regulation through phosphorylation of DNMT1 at S143, which disrupts its interaction with replication-associated proteins such as PCNA and UHRF1 and can thereby alter DNA methylation dynamics [63,64,65]. However, most evidence for this mechanism derives from non-cardiovascular systems, and its relevance in cardiovascular tissues remains incompletely characterized. Experimental studies in other model systems further suggest that AKT signaling may influence additional chromatin-modifying enzymes, including modulation of DNMT3a activity through glycogen synthase kinase-3 (GSK3) inhibition and phosphorylation-dependent regulation of histone modifiers such as EZH2 and KDM5A, thereby affecting histone methylation and transcriptional activation [66,67,68]. AKT signaling has also been reported to influence histone acetylation through phosphorylation of transcriptional coactivators such as p300/CBP, which can alter recruitment of transcription factors and chromatin accessibility [69,70]. While these mechanisms illustrate potential routes through which metabolic signaling pathways intersect with chromatin regulation, their functional relevance in cardiovascular tissues remains incompletely defined.

In contrast, activation of PI3K–AKT signaling downstream of estrogen receptor pathways is well established in cardiovascular biology. Estrogen–ER signaling in cardiomyocytes mitigates angiotensin II-induced ROS production via PI3K/AKT and p38 MAPK signaling, thereby exerting anti-apoptotic and anti-hypertrophic effects [71]. Similarly, GPER1 expressed in endothelial cells and cardiomyocytes mediates estrogen-induced cardioprotection through rapid non-genomic activation of PKB/AKT and MAPK signaling pathways [72,73] (Figure 2).

Given estrogen’s profound influence on epigenetic regulation, including its ability to modulate DNA methylation, histone modifications, and chromatin remodeling, understanding these interactions provides a promising avenue for the development of targeted therapies aimed at mitigating cardiovascular risks in postmenopausal women. The following sections delve deeper into estrogen-mediated epigenetic modifications and their implications for cardiometabolic health.

## 7. Role of DNA Methylation

Epigenetic synchronization of gene networks is essential for proper transcriptional control, and alterations in DNA methylation can disrupt this balance and promote disease. Beyond their classical genomic actions of E2/ER on EREs, ERs also regulate gene expression by influencing DNA methylation at transcription factor binding sites, cytosine and guanine-rich CpG islands, located within promoter or enhancer regions [74,75]. There are two functionally distinct classes of DNA methyltransferases (DNMTs): DNMT1 and DNMT3. DNMT1 preserves DNA methylation during replication [76]. DNMT3 comprises DNMT3a, DNMT3b, and DNMT3l; DNMT3a and DNMT3b catalyze de novo methylation [77], while DNMT3l does not bind DNA but interacts with DNMT3 proteins to enhance their activity [78,79]. DNA methylation exerts site-dependent effects: methylation near transcription start sites typically represses transcription, whereas methylation within gene bodies can facilitate transcriptional elongation [80].

**Figure 3:**
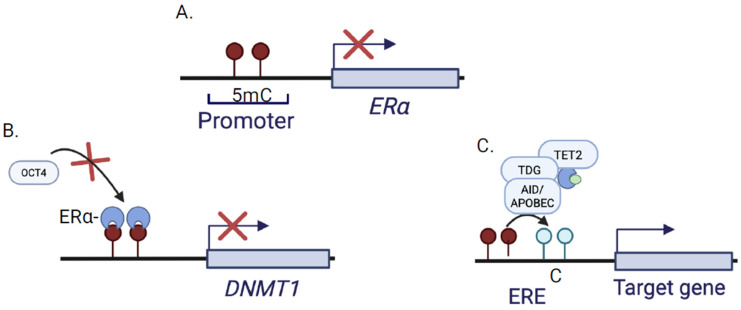
Reciprocal Interaction between estrogen receptor (ERα) signaling and DNA methylation: (**A**) Methylation-dependent repression of the *ESR1* gene encoding (ERα). Hypermethylation of CpG sites in the ERα promoter is associated with chromatin compaction and transcriptional silencing. (**B**) ERα-mediated passive DNA demethylation. Ligand-activated ERα has been reported to downregulate DNA methyltransferase 1 (DNMT1), the maintenance DNA methyltransferase. Reduced DNMT1 activity limits copying of 5-methylcytosine (5mC) during DNA replication, leading to progressive loss of methylation across cell divisions. (**C**) Estradiol has been reported to facilitate active demethylation at ER target loci. Upon estradiol (E2) binding, ER complexes occupy estrogen response elements (EREs) and recruit enzymes capable of catalysing removal of methyl marks. ER-bound ten-eleven translocase 2 (TET2) oxidizes 5mC to 5-hydroxymethylcytosine (5hmC); thymine DNA glycosylase (TDG) then excises the modified base, and the resulting abasic site is repaired to unmodified cytosine. ER can also recruit activation-induced cytidine deaminase (AID) and apolipoprotein B mRNA editing enzyme catalytic polypeptide-like (APOBEC) enzyme, which convert 5mC/5hmC to uracil-containing intermediates that are processed by base-excision repair and replaced with cytosine. These pathways illustrate how ER signaling can both respond to and actively remodel DNA methylation at its target genes. Panels depict established mechanisms reported in the literature, while the integration of passive and active demethylation pathways under ER control represents a conceptual framework summarizing current evidence.

Methylation-dependent ERα inactivation has been documented in vascular smooth muscle cells and is proposed to contribute to atherogenesis and cardiovascular aging, though direct validation in human atherosclerotic plaque tissue remains limited [81]. (Figure 3A). Reduced ERα expression may contribute to the limited efficacy of estrogen replacement therapy in postmenopausal women, including the outcomes reported in the HERS trial [81]. Promoter hypermethylation of ERα in vascular smooth muscle cells (VSMCs) under hyperinsulinemic conditions results from elevated DNMT activity, decreasing ERα expression and promoting atherosclerotic progression [82]. VSMCs are major sources of ROS in the vasculature, contributing to advanced atherosclerosis. Since ERα inhibits VSMC proliferation under high-glucose conditions, loss of ERα removes this inhibitory brake and thereby promotes atherosclerotic progression [83]. Conversely, ERα can also regulate the DNA methylation machinery itself. The interplay between the octamer-binding transcription factor (OCT4) and ERα at the ERE regulates DNMT1 expression [84]. OCT4 cannot promote DNMT1 expression in ERα-positive breast cancers because ERα occupies the ERE sequence in its promoter, thereby repressing DNMT1 transcription [84] (Figure 3B). Reduced DNMT1 levels impair maintenance methylation during replication, contributing to passive DNA demethylation. Intriguingly, E2 is a key regulator of both passive (Figure 3B) and active DNA demethylation (Figure 3C), in part by recruiting TET2/TDG demethylation machinery to ER-bound EREs.

Furthermore, the E2/ER module reduces methylation of autophagic genes, delaying cardiac aging and lowering cardiovascular disease risk in women [85]. Emerging evidence highlights cross-talk between non-coding RNAs and epigenetic regulation, as microRNA-152 is reduced in atherosclerosis, resulting in loss of DNMT inhibition, ERα promoter hypermethylation, and subsequent ERα downregulation [86].

## 8. Role of Histone Methylation

Histones form the protein core of nucleosomes and are critical regulators of chromatin accessibility and transcription. The extended N-terminal tails of histones H3 and H4 undergo multiple post-translational modifications, among which methylation and acetylation are particularly influential [87]. Histone methyltransferases regulate histone methylation by transferring methyl groups to lysine residues using S-adenosyl methionine as a substrate. Key histone methylation marks include H3K4, H3K9, and H3K27. While H3K9 and H3K27 methylation are linked to transcriptional repression, H3K4 methylation is associated with transcriptional activation. Higher-order methylation such as H3K9me2/3 promotes heterochromatin formation and transcriptional silencing [88]. In contrast, H3K4 methylation is enriched at transcriptionally active promoters and enhancers, with H3K4me1 particularly marking enhancer regions. H3K4me1 correlates with H3K27ac or H3K27me3, denoting active or inhibitory enhancers, respectively [89]. Additionally, arginine methylation on histones H3/H4 also promotes transcriptional activation, whereas its absence is linked to repression [90]. ER interactions with histone-modifying enzymes are well established in breast cancer contexts, where ERs recruit MLL2 to promote transcription through H3K4 methylation; whether analogous ER–MLL interactions operate in cardiac or vascular cells remains an open question [91]. The related methyltransferase MLL4 protects the heart from pressure-overload-induced hypertrophy and failure [92], raising the question of whether ER–MLL4 interactions influence cardiac remodeling.

**Figure 4:**
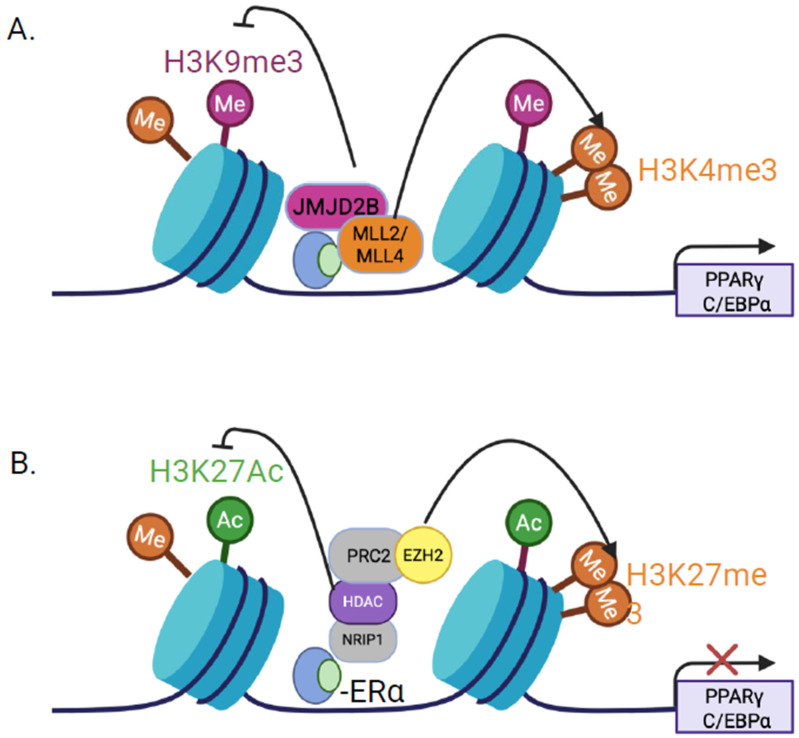
Epigenetically regulated poised chromatin-state regulates target gene expression levels: (**A**) Liganded estrogen receptor alpha (ERα) recruits histone lysine demethylase, JMJD2B, along with members of the SET-domain histone methyltransferases (MLL2/MLL4) facilitating removal of the repressive mark H3 lysine 9 trimethylation (H3K9me3) and promoting an active chromatin configuration characterized by H3 lysine 4 trimethylation (H3K4me3). (**B**) In alternative context, liganded ERα may recruit coregulators such as nuclear receptor interacting protein 1 (NRIP1), which facilitates the association of histone deacetylases (HDACs). HDAC activity removes H3 lysine 27 acetylation (H3K27ac), reducing chromatin accessibility. ERα-bound complexes can subsequently recruit the polycomb repressive complex 2 (PRC2), including enhancer of zeste homolog 2 (EZH2), which catalyze H3 lysine 27 trimethylation (H3K27me3), reinforcing a repressive chromatin state. These opposing histone-modifying pathways illustrate how ERα promote or restrain adipogenic gene expression (e.g., *PPARγ* and *C/EBPα*) depending on the chromatin context. Established enzymatic activities (e.g., JMJD2B-mediated H3K9 demethylation and EZH2-mediated H3K27 methylation) are shown, whereas the integration of these pathways into a context-dependent regulatory switch represents a conceptual framework.

Aberrant histone modifications contribute to cardiovascular disease. The histone lysine demethylase JMJD2B (KDM4B) promotes vascular calcification by altering chromatin accessibility at the STAT3-binding site of the RUNX2 promoter, reducing H3K9me3 [93]. Given that KDM4B also targets PPARγ and C/EBPα promoters to promote adipogenesis, ERα may regulate adipogenesis via a KDM4B/MLL axis [22] (Figure 4A). The polycomb methyltransferase EZH2 integrates IL-1β and TGFβ2 signaling pathways, which downregulate EZH2 and the repressive H3K27me3 mark at the TAGLN promoter [94], thereby enhancing TAGLN/SM22α expression, crucial for vascular smooth muscle cell function [94] (Figure 4B). Papait et al. (2017) proposed that histone methyltransferase G9a and transcription factor MEF2C cooperatively repress a subset of genes critical for cardiac homeostasis. Their study identifies H3K9me2 as an epigenetic mark for MEF2C-driven repression in unstressed hearts and suggests synergistic mechanism of gene silencing [95]. Given that E2/ER signaling via PI3K/AKT phosphorylates EZH2 at S21, reducing repressive H3K27me3 levels [96], further studies should explore whether non-genomic estrogen signaling modulates cardiac function through similar histone-based mechanisms. Similarly, Smyd1, a muscle-specific histone methyltransferase, is essential for cardiac homeostasis, as its knockout in adult mice leads to hypertrophy and heart failure [97]. Although direct evidence of ER-mediated regulation of JMJD2B and Smyd1 in cardiomyocytes is lacking, these findings suggest histone methylation significantly contributes to cardiovascular disease pathogenesis.

## 9. Role of Histone Acetylation

Histone acetylation is a dynamic epigenetic mark critical for transcriptional regulation, coordinated by histone acetyltransferases (HATs) and histone deacetylases (HDACs). p300/CBP, an atypical HAT lacking a conserved catalytic motif, functions as a transcriptional adaptor that recruit other HATs, such as p300/CBP-associated factor (PCAF) [98]. HDACs are grouped into four classes: Class I (HDAC1, 2, 3, and 8), Class IIa (HDAC4, 5, 7, and 9), Class IIb (HDAC6 and HDAC10), Class III (Sirtuin family SIRT1-SIRT7), and Class IV (HDAC11). Acetylation marks such as H3K9ac and H3K27ac are key indicators of active chromatin. Aberrant histone acetylation contributes to both cancer [99] and cardiovascular disease [100], and HDACs play essential roles in cardiac hypertrophy and heart failure [101].

In models of postmenopausal metabolic syndrome, cardiac apoptosis is associated with SIRT1 downregulation and increased global H3 acetylation in the aorta and heart. Estrogen receptor signaling partially reverses these effects by suppressing angiotensin II-induced contractions, restoring SIRT1 expression, and reducing H3 acetylation [102]. SIRT1 overexpression in diabetic rat hearts improves cardiac function, reduces infarct size, and mitigates oxidative stress through endothelial nitric oxide synthase (eNOS) activation [103]. HDAC3 counteracts this by deacetylating eNOS, thereby reducing endothelial NO production [104]. In cardiac hypertrophy, Class II HDACs (HDAC4, 5, and 9) exert protective, anti-hypertrophic effects [105,106], whereas Class I HDAC2 promotes hypertrophic remodeling. Estrogen receptor activation attenuates fibrosis and apoptosis in pressure-overload models, delaying progression to heart failure [107,108].

ERβ activation inhibits angiotensin II-induced HDAC2 activation, suppressing hypertrophic gene expression, while stabilizing HDAC4 and HDAC5 in the nucleus to repress hypertrophic transcription programs [109]. In contrast, ERα signaling enhances vascular endothelial growth factor (VEGF) expression in HDAC5/9-deficient models, improving post-MI survival [110]. These divergent receptor-specific roles highlight the need to further clarify how ERα and ERβ interact with HDACs and HATs to shape cardiac remodeling and cardiovascular outcomes in postmenopausal women.

### 9.1. Estrogen-Mediated Epigenetic Regulation in Postmenopausal Women

Premenopausal women exhibit a lower incidence of CVD compared with age-matched men, largely owing to estrogen’s cardioprotective actions. SIRT1, an estradiol-induced histone deacetylase, confers cardiomyocyte protection against angiotensin II-induced damage in postmenopausal metabolic syndrome models [102]. Estrone (E1), present at lower concentration in men and postmenopausal women compared to premenopausal women [111], has demonstrated mixed cardiovascular effects [112,113]. Some evidence suggests estriol (E3) enhances endothelial function by increasing nitric oxide (NO) bioavailability, potentially explaining its anti-atherosclerotic role in postmenopausal women [114]. Given NO’s interplay with HDACs, further research is warranted to delineate its epigenetic regulatory mechanisms.

Estrogen deficiency after menopause contributes to increased adiposity, particularly visceral adipose tissue (VAT), which contributes to metabolic dysfunction. Menopausal hormone therapy significantly reduces VAT and fat mass in postmenopausal women [115,116]. Mechanistically, estrogen represses adipogenesis by downregulating key differentiation factor while promoting osteogenesis in mesenchymal stem cells. Erα-mediated repression of *PPARγ* and *C/EBPα* through recruitment of EZH2 and deposition of the repressive H3K27me3 mark restricts adipocyte formation [117] (Figure 4B). These findings suggest that estrogen modulates adiposity through epigenetic mechanisms that influence PPAR-dependent lipid metabolic pathways.

Heart failure with preserved ejection fraction (HFpEF) disproportionately affects postmenopausal women, with estrogen depletion contributing to its progression [118]. Compared with men, women with HFpEF exhibit greater left ventricular hypertrophy, diastolic dysfunction, and a higher prevalence of atrial fibrillation [119,120]. Estrogen regulates eNOS, a key factor in cGMP-PKG signaling, which is compromised in HFpEF [121]. Reduced NO availability contributes to vascular stiffness and impaired myocardial relaxation, hallmarks of HFpEF, and these abnormalities are particularly prominent in postmenopausal women [119,122]. Therapies targeting natriuretic peptide–cGMP signaling, including phosphodiesterase-9A (PDE9A) inhibition, may improve metabolic and cardiac function by restoring downstream PKG activity [118].

Growing evidence highlights PPARα–ER crosstalk as a critical determinant of HFpEF progression, particularly in obesity-related phenotypes. PPARα activation enhances fatty acid oxidation, mitochondrial biogenesis, and metabolic efficiency, which are impaired in cardiometabolic syndrome (CMS) and HFpEF [123]. Notably, PDE9A inhibition reduces fat mass, myocardial hypertrophy, and fibrosis via a PPARα-mediated pathway, suggesting that enhancing PPARα restores myocardial energy metabolism and cardiac function in postmenopausal obesity [123]. These PPARα-driven metabolic benefits exhibit sexual dimorphism, with pronounced effects in males and ovariectomized females, indicating that estrogen modulates PPARα-driven lipid oxidation and mitochondrial function [123]. Given these findings, further mechanistic investigation into PPARα–ER interactions is warranted. Precision-targeted therapies that combine PPARα agonists with PDE9A inhibitors may offer synergistic benefits for postmenopausal women by simultaneously addressing hormone-dependent metabolic alterations and cardiovascular dysfunction [118].

### 9.2. Pharmacological Interventions for Mitigating Cardiometabolic Risks

Cardiometabolic diseases remain a leading cause of mortality in women. Compared with men, cardiovascular disease (CVD) in women often manifests at an older age, with risk rising sharply during the menopausal transition due in part to declining estrogen levels and redistribution of adipose tissue [124,125]. While the preceding sections outline estrogen-dependent chromatin regulation as a potential upstream contributor to this increased risk, current clinical management primarily targets downstream manifestations of disease. Importantly, several pharmacologic agents, including statins, GLP-1 receptor agonists, SGLT2 inhibitors, and hormone replacement therapy, have been reported in preclinical studies to influence epigenetic pathways, providing a potential mechanistic bridge between conventional therapeutics and chromatin-level dysregulation. This section reviews these strategies within that integrated framework.

Exercise, diet-derived metabolites, and metabolic therapeutics may converge on chromatin-modifying enzymes by altering cellular metabolic state. For example, exercise-induced energetic stress activates AMP-activated protein kinase (AMPK) signaling and can increase NAD^+^ availability, thereby promoting activation of NAD^+^-dependent deacetylases such as SIRT1 that influence chromatin remodeling and transcriptional regulation [124]. In parallel, nutrient-sensitive metabolites including acetyl-CoA and S-adenosylmethionine (SAM) couple cellular metabolic flux to histone acetylation and DNA or histone methylation reactions, linking metabolic state directly to epigenetic regulation of gene expression [125,126,127,128]. Metabolic drugs such as metformin similarly activate AMPK signaling pathways, further illustrating how cardiometabolic therapeutics may influence chromatin-modifying enzymes through metabolic signaling networks [128]. Consistent with this concept, acute exercise has been shown to induce epigenetic remodeling of metabolic gene promoters in human skeletal muscle [129]. Although most mechanistic evidence derives from metabolic tissues rather than cardiovascular systems, these observations suggest that metabolic and lifestyle interventions may intersect with estrogen receptor signaling pathways to influence estrogen-sensitive epigenetic regulation of cardiometabolic gene programs.

## 10. Lipid-Lowering Medications

Hyperlipidemia is a major contributor to CVD in postmenopausal women. Statins inhibit HMG-CoA reductase, reducing endogenous cholesterol synthesis and upregulating LDL receptors to enhance LDL clearance [130,131]. Statin therapy reduces adverse cardiovascular events even in low- and intermediate-risk patients [132,133]. Emerging preclinical evidence also suggests that statins can modulate DNA methylation and histone acetylation patterns in vascular endothelial cells, potentially contributing to anti-inflammatory effects through chromatin-level mechanisms beyond LDL reduction [134]. Ezetimibe inhibits Niemann-Pick C1-like 1 (NPC1L1) protein to prevent dietary cholesterol absorption and is particularly beneficial in statin-intolerant individuals [131,135]. PCSK9 inhibitors, such as monoclonal antibodies, enhance LDL receptor availability, further reducing LDL levels [136]. The ODYSSEY LONG-TERM trial demonstrated their efficacy in high-risk patients and highlighted their value as add-on therapy to statins [137]. Bempedoic acid, which inhibits ATP-citrate lyase, provides a statin-independent LDL-lowering approach with minimal risk of myopathy [138]. The CLEAR Outcomes trial showed that bempedoic acid improves cardiovascular endpoints in statin-intolerant individuals [139]. Fibrates activate PPARα, promoting lipoprotein lipase activity and enhancing fatty acid oxidation, making them useful in patients with hypertriglyceridemia [140]. Although the PROMINENT trial found that pemafibrate did not reduce major cardiovascular events, it effectively lowered triglyceride and VLDL cholesterol levels [141].

## 11. Cardioprotective Diabetic Medications

The incidence of T2DM increases in postmenopausal women, further elevating cardiovascular risk. Glucagon-like peptide-1 (GLP-1) receptor agonists improve glycemic control by stimulating insulin secretion and suppressing glucagon release [142,143]. Several GLP-1 receptor agonists have demonstrated reductions in major adverse cardiovascular events, making them an important therapeutic option for postmenopausal women with T2DM and elevated cardiovascular risk [143]. Preclinical studies further suggest that GLP-1 receptor agonists reduce NLRP3 inflammasome activation in macrophages and may modulate histone acetylation states in metabolically active tissues, linking some of their cardiometabolic benefits to immune–epigenetic mechanisms [144].

Sodium-glucose co-transporter-2 (SGLT2) inhibitors promote glycosuria to reduce hyperglycemia and provide additional cardioprotective benefits, including lowering blood pressure and reducing hospitalizations for heart failure [145]. Agents such as Empagliflozin and dapagliflozin have shown efficacy in reducing cardiovascular mortality in postmenopausal women with diabetes and heart disease [146]. Although there are a number of glucose-lowering medications for T2DM, the American College of Cardiology recommends GLP-1 agonists and SGLT2 inhibitors specifically for their proven cardioprotective qualities [147,148]. Additionally, SGLT2 inhibitors have been reported in preclinical models to influence chromatin-associated pathways, including reduced repressive histone marks and increased sirtuin (SIRT1/SIRT3) activity in cardiac and renal tissues, suggesting a potential epigenetic component to metabolic gene network remodeling [149].

## 12. Antihypertensive Medications

Postmenopausal women frequently experience hypertension due to arterial stiffening and increased sympathetic nervous system activity. The renin–angiotensin–aldosterone system is a major therapeutic target. Angiotensin-converting enzyme (ACE) inhibitors and angiotensin II receptor blockers (ARBs) mitigate vasoconstriction and sodium retention, improving endothelial function [150]. In women with both hypertension and T2DM, these agents also decrease proteinuria and slow the progression of diabetic kidney disease [151]. Diuretics are foundational in hypertension management. Thiazide diuretics inhibit sodium reabsorption at the distal convoluted tubule, while loop diuretics inhibit the Na^+^/K^+^/2Cl^−^ transporter at the loop of Henle, effectively reducing fluid retention and lowering blood pressure [152]. Thiazides are particularly useful in hypertensive postmenopausal women with coexisting osteoporosis, as they lower urinary calcium excretion and may reduce fracture risk.

## 13. Hormone Replacement Therapy (HRT)

Hormone replacement therapy remains a complex yet important consideration in cardiovascular health management for postmenopausal women. Early observational studies suggested a protective role of estrogen against CVD; however, large randomized trials, including the Women’s Health Initiative (WHI) and the Heart and Estrogen/Progestin Replacement Study (HERS), identified increased risks of thrombotic events and coronary artery disease when HRT was initiated in older women or long after menopause [153,154,155]. Oral estrogen may elevate thrombosis risk by increasing von Willebrand factor and multiple coagulation factors [156,157,158]. In contrast, transdermal estradiol with micronized progesterone demonstrates a more favorable safety profile, with a lower risk of venous thromboembolism and fewer adverse cardiovascular effects [159]. The timing hypothesis suggests that early HRT initiation (<60 years or within 10 years of menopause) may confer cardioprotective effects, while late initiation elevates risk [160]. From an epigenetic perspective, progressive silencing of ER-responsive programs—including promoter methylation changes reported in vascular cells—has been proposed as one mechanism that could reduce tissue responsiveness to estrogen with delayed initiation, providing a chromatin-level rationale that warrants longitudinal validation in human cardiovascular tissues.

The route and formulation influence outcomes, with transdermal and vaginal estrogen demonstrating fewer adverse effects than oral preparations [161]. Current guidelines recommend HRT at the lowest effective dose for symptomatic relief while considering cardiovascular risk factors [154].

### Lifestyle Interventions for Cardiometabolic Health

Pharmacological and lifestyle interventions are critical in managing cardiometabolic risks in postmenopausal women. While lipid-lowering, antidiabetic, and antihypertensive medications provide targeted risk reduction, sustained benefits also depend on non-pharmacological measures, including dietary modification, regular physical activity, psychosocial support, and moderation of smoking and alcohol use.

## 14. Dietary Interventions

The Mediterranean and Dietary Approaches to Stop Hypertension (DASH) diets improve cardiovascular health by reducing inflammation, oxidative stress, and insulin resistance. Rich in polyphenols, omega-3 fatty acids, and fiber, these diets modulate lipid metabolism and enhance nitric oxide bioavailability, counteracting postmenopausal metabolic decline [162,163,164,165,166]. Polyphenols influence DNA methylation and histone modifications, regulating genes involved in glucose and lipid metabolism [162]. Omega-3 fatty acids reduce triglycerides and increase HDL cholesterol, while fiber slows glucose absorption, stabilizing blood sugar levels [167,168,169]. Large scale epidemiological studies support the protective role of these diets against CVD. The PREDIMED trial found that adherence to a Mediterranean diet supplemented with extra-virgin olive oil or nuts reduced major cardiovascular events by 30% in high-risk individuals over a 4.8-year median follow-up [170]. Globally suboptimal diet contributes to 11 million deaths annually, with high sodium intake, low intake of whole grains, and insufficient fruit intake as major drivers of cardiovascular mortality [171]. In the Nurses’ Health Study and Health Professionals Follow-up Study, higher scores on healthy dietary indices (HEI-2005 and AHEI-2010) were associated with approximately 25–30% lower risk of coronary heart disease [172]. and adherence to a DASH-style dietary pattern was associated with around 20–25% lower risk of coronary heart disease [173].

## 15. Heart Failure (HF) Trials and Mechanisms of Action

Several clinical trials have examined the effect of these diets on heart failure (HF) outcomes. The DASH-Sodium Trial demonstrated significant reductions in systolic (6.7 mmHg) and diastolic (3.5 mmHg) blood pressure, with the greatest benefit observed in individuals with hypertension [174]. The MIND diet trial, which integrates Mediterranean and DASH components, reported improved vascular function and slower cognitive decline, indirectly supporting HF risk reduction [175]. A recent meta-analysis in women reported that higher adherence to the Mediterranean diet was associated with a 24% lower incidence of cardiovascular disease and a 23% lower risk of total mortality [176]. The Women’s Health Initiative (WHI) Cohort Study, (93,676 postmenopausal women) higher Alternate Healthy Eating Index (AHEI) diet quality was associated with a 23% lower risk of incident cardiovascular disease and a 30% lower risk of incident heart failure [177]. A meta-analysis of prospective cohorts showed that high adherence to a DASH-style diet was associated with a 29% lower risk of incident heart failure in the general population [178]. Omega-3 fatty acids lower triglyceride levels by 12–30%, increase HDL cholesterol, and reduce systemic inflammation, contributing to improved cardiac function and reduced HF risk [179]. Dietary fiber further attenuates postprandial glucose excursion by 25–30%, and enhances gut microbiota diversity, collectively improving metabolic stability [169]. These mechanistic benefits reinforce the therapeutic role of Mediterranean and DASH-based dietary patterns in cardiovascular health, and HF prevention.

## 16. Physical Activity

Aerobic exercise improves endothelial function by increasing nitric oxide production, reducing arterial stiffness, and lowering blood pressure [180,181]. Resistance training is essential for preserving muscle mass, counteracting sarcopenia, and improving insulin sensitivity [182,183]. Regular physical activity also reduces systemic inflammation, lowering C-reactive protein and interleukin-6 levels [184]. The American Heart Association (AHA) 2021 guidelines recommend at least 150 min per week of moderate-intensity aerobic activity, along with muscle-strengthening exercises on two or more days per week [185]. Aerobic exercise has been associated with altered DNA methylation at inflammatory and metabolic loci and increased sirtuin activity, supporting the concept that exercise functions as a systemic epigenetic modulator that may partially offset estrogen-dependent chromatin dysregulation after menopause [186,187,188].

Achieving these activity levels has been associated with approximately a 22–31% reduction in cardiovascular disease risk in large prospective cohort studies [189,190]. Specifically, meeting guideline-level moderate physical activity is linked to a 22–25% lower risk of cardiovascular mortality, while engaging in vigorous activity at recommended levels confers an approximately a 31% reduction in cardiovascular mortality [189]. The European Society of Cardiology (ESC) guidelines reinforce this, linking high physical activity to a 20–30% lower risk of major cardiovascular events [191]. Clinical trials further support these benefits: a meta-analysis of 28 trials showed that supervised aerobic training leads to a 3.5 mmHg reduction in systolic blood pressure and a 2.5 mmHg reduction in diastolic blood pressure [192]. Additionally, resistance training has been found to improve insulin sensitivity by up to 24% in individuals with metabolic syndrome [193]. The combined effects of aerobic and resistance training underscore the critical role of physical activity in cardiovascular disease prevention and overall metabolic health.

## 17. Smoking and Alcohol Moderation

Smoking accelerates oxidative stress, endothelial dysfunction, and arterial stiffness, significantly increasing cardiovascular risk in postmenopausal women [194,195]. In the large prospective Million Women Study cohort studies [196] lifelong smokers have roughly a threefold higher risk of cardiovascular mortality (CVD) compared with never-smokers. Even light smoking (1–4 cigarettes/day) markedly increases coronary heart disease (CHD) risk [197]. In the Women’s Health Initiative, smoking cessation was associated with substantial reductions in coronary heart disease (CHD) risk. Compared with women who continued smoking, recent quitters had ~25% lower CHD risk, while long-term former smokers had approximately 60% lower risk [198].

Excessive alcohol consumption exacerbates systemic inflammation, raises triglyceride levels, and contributes to metabolic dysregulation, outweighing any potential cardiovascular benefits of low-to-moderate intake. Meta-analytic data show that heavy drinking (>60 g/day) is associated with a substantially higher risk of total and hemorrhagic stroke, whereas light-to-moderate intake is associated with a lower risk of total and ischemic stroke compared with abstention [199]. In contrast, low-to-moderate average alcohol intake without binge-drinking episodes shows neutral to modestly lower risk of ischemic heart disease, while heavier and/or binge patterns are linked to increased cardiovascular events and mortality [200,201]. A large Kaiser Permanente cohort study of more than 432,000 adults further reported that women exceeding recommended weekly alcohol limits (≥8 drinks/week) had approximately 33–51% higher risk of incident coronary heart disease compared with women who drank less [202]. Collectively, these findings support moderation of alcohol intake as a core lifestyle target for reducing cardiovascular risk, particularly in women.

## 18. Conclusions

**Figure 5:**
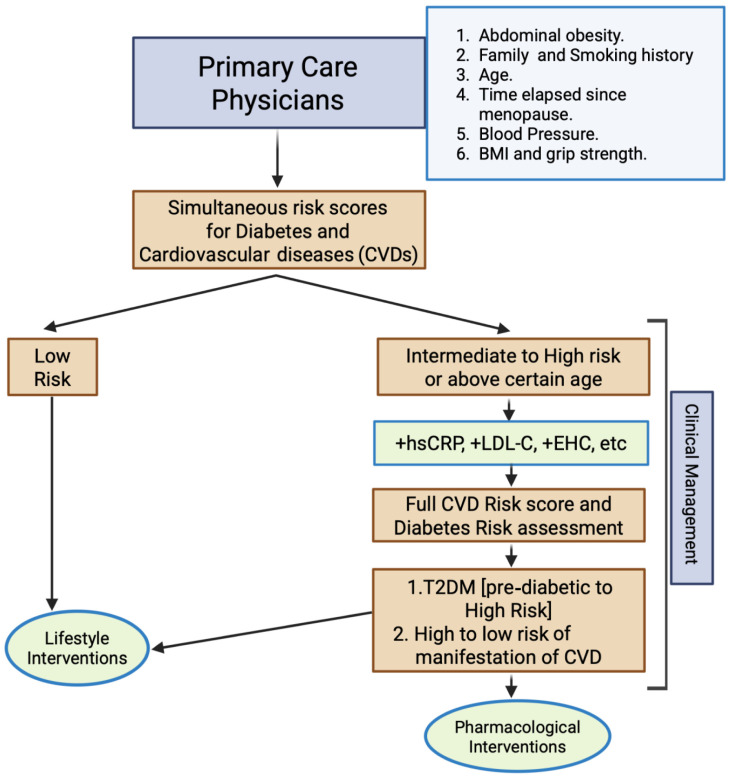
Risk assessment framework for postmenopausal women’s health management: This schematic outlines a stepwise approach for evaluating cardiometabolic risk in postmenopausal women and guiding clinical management. Primary care physicians integrate core risk determinants, including abdominal obesity, family history and smoking status, blood pressure, age, time since menopause, body mass index (BMI), and functional indicators such as grip strength to determine an initial risk category. Women identified as low-risk are directed toward lifestyle-focused strategies emphasizing diet, physical activity, and weight management. Individuals falling into an intermediate- or high-risk category, or those above a certain age threshold, may undergo additional biomarker assessment (e.g., high-sensitivity C-reactive protein [hs-CRP], low-density lipoprotein cholesterol [LDL-C], and metabolic indices such as the euglycemic–hyperinsulinemic clamp [EHC], where applicable). Comprehensive evaluation of cardiovascular disease (CVD) and type 2 diabetes (T2DM) risk then informs targeted interventions, ranging from intensified lifestyle modification to pharmacological therapy for prediabetes or elevated CVD risk. This integrated framework may support personalized decision-making aimed at reducing cardiometabolic burden and improving long-term health outcomes in postmenopausal women.

The postmenopausal transition represents a pivotal period in women’s cardiometabolic health. Estrogen withdrawal influences vascular biology, metabolic regulation, and inflammatory signaling, and may also alter chromatin states across multiple tissues. The framework presented here proposes that epigenetic remodeling constitutes a dynamic and tissue-specific regulatory interface linking hormonal loss to cardiometabolic dysfunction. However, this model remains incompletely validated in human cardiovascular tissues and should be interpreted as a hypothesis-driven framework supported primarily by preclinical and associative evidence. Rather than serving as a singular causal pathway, epigenetic modulation should be viewed as one mechanistic layer within a multifactorial process that includes vascular aging, receptor expression changes, metabolic stress, and immune activation. Accordingly, comprehensive risk assessment and management strategies remain essential. The integrated clinical framework illustrated in Figure 5 outlines pharmacologic and lifestyle interventions—including hormone replacement therapy, lipid-lowering agents, cardioprotective antidiabetic therapies, structured exercise programs, and heart-healthy dietary patterns—that collectively address cardiometabolic risk in postmenopausal women. While several of these interventions have demonstrated epigenetic modulatory effects in preclinical studies, no chromatin-targeted therapies are currently approved for cardiometabolic disease. Future research integrating longitudinal human epigenomic profiling, cell-type-specific analysis, and translational therapeutic studies will be essential to determine how estrogen-dependent chromatin regulation may ultimately inform precision strategies for optimizing cardiovascular and metabolic health in postmenopausal women.

## Figures and Tables

**Table 1:** STRAW staging system in women; ⬆ Elevated hormonal levels; **⬇** low hormonal levels; follicle-stimulating hormone (FSH); anti-Müllerian hormone (AMH).

Stages	Reproductive			Menopausal Transition		Postmenopause	
	Early	Peak	Late	Early	Late	Early	Late
Duration	Variable			Variable		Until demise	
Menstrual cycle	Variable to Regular	Regular		Variable cycle length (>7 days difference in length of consecutive cycles)	>=2 skipped cycles and an interval of amenorrhea (>=60 days)	none	
**Endocrine**							
FSH	Normal		⬆				
AMH	Normal		**⬇**	**⬇**	⬆ Undetectable levels ⬆		
Inhibin B	Normal		**⬇**	**⬇**	Undetectable levels		
Estradiol	Normal				**⬇**	**⬇**	

**Table 2:** Tissue-Specific Estrogen Receptor Subtype, Epigenetic Mechanism, and Level of Evidence in Cardiometabolic Disease. Legend: ER, estrogen receptor; DNMT, DNA methyltransferase; SIRT1, sirtuin 1; HDAC, histone deacetylase; EZH2, enhancer of zeste homolog 2; H3K27me3, trimethylation of histone H3 at lysine 27; PPARγ, peroxisome proliferator-activated receptor gamma; C/EBPα, CCAAT/enhancer-binding protein alpha; NLRP3, NLR family pyrin domain-containing 3; eNOS, endothelial nitric oxide synthase; GPER, G-protein-coupled estrogen receptor; CVD, cardiovascular disease. Evidence levels reflect the strongest available data for each mechanism; most findings await direct validation in human cardiovascular tissues.

Tissue/Cell Type	ER Subtype	Epigenetic Mechanism	Functional Outcome	Evidence Level
Vascular smooth muscle cell (VSMC)	ERα	DNMT-mediated ERα promoter hypermethylation; miR-152–DNMT axis	↑ VSMC proliferation; atherosclerotic progression	In vitro; human associative
Cardiomyocyte	ERα, ERβ	SIRT1 induction; H3 acetylation reduction; HDAC2 suppression via ERβ	Anti-apoptotic; anti-hypertrophic; cardioprotection in pressure overload	Animal models; in vitro
Vascular endothelial cell	ERα, GPER	AKT-mediated EZH2 phosphorylation (↓ H3K27me3); H3K56 acetylation via p300/CBP	Angiogenesis; eNOS activation; anti-inflammatory	In vitro; animal models
Hepatocyte	ERα	ERα regulation of DNMT activity; triglyceride gene methylation	Lipid homeostasis; prevention of hepatic steatosis	Animal models; in vitro
Adipocyte/Mesenchymal stem cell	ERα	EZH2 recruitment; H3K27me3 at PPARγ/C/EBPα promoters	Repression of adipogenesis; promotion of osteogenesis	Animal models; in vitro
Macrophage/Immune cell	ERα, ERβ	NLRP3 acetylation/deacetylation; histone acetylation at inflammatory gene loci	Modulation of inflammasome activation; insulin sensitivity	In vitro; animal models
Cardiac aging/Autophagy	E2/ER (general)	Reduced methylation of autophagic gene promoters	Delayed cardiac aging; reduced CVD risk in women	Animal models

## Data Availability

No new data were created or analyzed in this study.
